# Supplementary material for: EnzML: multi-label prediction of enzyme classes using InterPro signatures
Source: BMC Bioinformatics. 2012 Apr 25;13:61. doi: 10.1186/1471-2105-13-61 (PMC3483700; doi:10.1186/1471-2105-13-61)
Supplement: Addtional file 5 — The Java code to format the data files, evaluate and predict. The file enzml_java_code.tar.gz contains the Java code used to format database data to ARFF and XML formats, to execute cross and train-test (jackknife) evaluations and to record evaluation results to database. More information is included in the readme.txt file and the Javadoc files. The code can be used with a MySQL database. To use a different database software, other JDBC drivers might be required. [file 1471-2105-13-61-S5.gz › java_code/utils/doc/edu/cornell/lassp/houle/RngPack/Ranmar.html]

Ranmar


---


|  |  |  |
| --- | --- | --- |
| **Overview** \| **Package** \| **Class** \| **Use** \| **Tree** \| **Deprecated** \| **Index** \| **Help** | |  |
| **PREV CLASS**   NEXT CLASS | **FRAMES**    **NO FRAMES**     **All Classes** |  |
| SUMMARY: NESTED \| FIELD \| CONSTR \| METHOD | DETAIL: FIELD \| CONSTR \| METHOD |  |


---


## edu.cornell.lassp.houle.RngPack Class Ranmar

```
java.lang.Object
  cern.colt.PersistentObject
      edu.cornell.lassp.houle.RngPack.RandomElement
          edu.cornell.lassp.houle.RngPack.RandomSeedable
              edu.cornell.lassp.houle.RngPack.Ranmar
```

**All Implemented Interfaces:**: java.io.Serializable, java.lang.Cloneable

---

``` public class Ranmar extends RandomSeedable ```

RANMAR is a lagged Fibonacci generator proposed by Marsaglia and
Zaman and is a good research grade generator. This version of RANMAR
is based on the paper by James, which is a good reference for the properties
of RANMAR and several other generators.
  
**REFERENCES:**   
F. James, Comp. Phys. Comm. **60** (1990) p 329-344   
and was originally described in   
G. Marsaglia, A. Zaman and W.-W Tsang, Stat. Prob. Lett
**9** (1990) p 35.  More
info .

Source code  is available.

**Version:**
:   1.0

**Author:**
:   Paul Houle  (E-mail: houle@msc.cornell.edu)

**See Also:**: Serialized Form

---

| **Field Summary** | |
| --- | --- |
| `static int` | `BIG_PRIME`             The 46,009,220nd prime number, he largest prime less than 9\*10^8^. |
| `static int` | `DEFSEED`             Default seed. |


**Constructor Summary**

`Ranmar()`
  
          Initialize Ranmar with a default seed taken from Marsaglia and Zaman's
paper.

`Ranmar(java.util.Date d)`
  
          Seed RANMAR from the clock.

`Ranmar(int ijkl)`
  
          Initialize Ranmar with a specified integer seed

`Ranmar(long ijkl)`
  
          Initialize Ranmar with a specified long seed


| **Method Summary** | |
| --- | --- |
| `java.lang.Object` | `clone()`             Returns a copy of the receiver; the copy will produce identical sequences. |
| `double` | `raw()`             Returns a uniformly distributed pseudo-random double in the range (0,1). |
| `void` | `raw(double[] d, int n)`             A version of the generator for filling arrays, inlined for speed |

**Methods inherited from class edu.cornell.lassp.houle.RngPack.RandomSeedable**

`ClockSeed, ClockSeed`

**Methods inherited from class edu.cornell.lassp.houle.RngPack.RandomElement**

`choose, choose, gaussian, gaussian, powlaw, raw, uniform`

**Methods inherited from class java.lang.Object**

`equals, getClass, hashCode, notify, notifyAll, toString, wait, wait, wait`

**Field Detail**

### BIG\_PRIME

```
public static int BIG_PRIME
```

:   The 46,009,220nd prime number, he largest prime less than
    9\*10^8^. Used as a modulus because this version of
    RANMAR needs a seed between 0 and 9\*10^8^ and
    `BIG_PRIME` isn't commensurate with any regular period.
    `BIG_PRIME= 899999963`

---


### DEFSEED

```
public static int DEFSEED
```

:   Default seed. `DEFSEED=54217137`


**Constructor Detail**

### Ranmar

```
public Ranmar()
```

:   Initialize Ranmar with a default seed taken from Marsaglia and Zaman's
    paper. Equivalent to `Ranmar(54217137).`

---


### Ranmar

```
public Ranmar(java.util.Date d)
```

:   Seed RANMAR from the clock.

    ```
     RandomElement e = new Ranmar(new Date());
    ```

    **Parameters:**: `d` - a Date object to seed Ranmar with, typically `new Date()`

---


### Ranmar

```
public Ranmar(int ijkl)
```

:   Initialize Ranmar with a specified integer seed

    **Parameters:**: `ijkl` - seed integer; Ranmar(int ijkl) takes uses ijkl modulus BIG\_PRIME as a seed for RANMAR.

---


### Ranmar

```
public Ranmar(long ijkl)
```

:   Initialize Ranmar with a specified long seed

    **Parameters:**: `ijkl` - seed long; Ranmar(long ijkl) takes uses ijkl modulus BIG\_PRIME as a seed for RANMAR.


**Method Detail**

### clone

```
public java.lang.Object clone()
```

:   Returns a copy of the receiver; the copy will produce identical
    sequences. After this call has returned, the copy and the receiver have
    equal but separate state.

    :   **Overrides:**: `clone` in class `PersistentObject`
    :   **Returns:**: a copy of the receiver.

---


### raw

```
public final double raw()
```

:   Returns a uniformly distributed pseudo-random double in the range (0,1).

    :   **Specified by:**: `raw` in class `RandomElement`
    :   **Returns:**: a pseudo random number in the range (0,1). **See Also:**: `RandomJava`

---


### raw

```
public final void raw(double[] d,
                      int n)
```

:   A version of the generator for filling arrays, inlined for speed

    :   **Overrides:**: `raw` in class `RandomElement`
    :   **Parameters:**: `d` - an array of doubles to be filled: `n` - size of the array


---


|  |  |  |
| --- | --- | --- |
| **Overview** \| **Package** \| **Class** \| **Use** \| **Tree** \| **Deprecated** \| **Index** \| **Help** | |  |
| **PREV CLASS**   NEXT CLASS | **FRAMES**    **NO FRAMES**     **All Classes** |  |
| SUMMARY: NESTED \| FIELD \| CONSTR \| METHOD | DETAIL: FIELD \| CONSTR \| METHOD |  |


---
